# Efficacy and safety of early administration of remdesivir in hemodialysis patients with COVID-19: A case report and literature review

**DOI:** 10.1097/MD.0000000000040650

**Published:** 2024-11-29

**Authors:** Nanako Oshiro, Takeshi Kinjo, Daigo Aharen, Yuki Kudo, Eisuke Katsuren, Kumiko Omine, Takuto Nakamura, Ryo Zamami, Akio Ishida, Kazuya Miyagi, Masashi Nakamatsu, Kazuko Yamamoto, Kenya Kusunose, Jiro Fujita, Yusuke Ohya, Kentaro Kohagura

**Affiliations:** a Department of Cardiovascular, University of the Ryukyus Graduate School of Medicine, Nephrology, and Neurology, Okinawa, Japan; b Department of Infectious, Respiratory, and Digestive Medicine, University of the Ryukyus Graduate School of Medicine, Okinawa, Japan; c University of the Ryukyus Hospital, Infection Control Team, Okinawa, Japan; d Dialysis Unit, University of the Ryukyus Hospital, Okinawa, Japan; e Respiratory Medicine, Ohama Daiichi Hospital, Okinawa, Japan.

**Keywords:** COVID-19, hemodialysis, remdesivir, SARS-CoV-2

## Abstract

**Rationale:**

Although the mortality of severe coronavirus disease 2019 (COVID-19) has decreased after the emergence of the Omicron variant, it remains high in patients on hemodialysis (HD). Remdesivir (RDV) is considered as the first line drug for hospitalized COVID-19 patients, however the evidence regarding the usage in HD patients is lacking because clinical trials of RDV have excluded HD patients for safety reasons. Thus, accumulation of knowledge on the regimen, efficacy, and tolerability of RDV in HD patients is important.

**Patient concerns:**

A nosocomial COVID-19 cluster was occurred from August 31 to October 12 in 2021 when the Delta variant was predominant. During the cluster, 11 health-care workers and 20 inpatients including 7 HD patients were infected with severe acute respiratory syndrome coronavirus 2 (SARS-CoV-2).

**Diagnoses:**

The diagnosis of COVID-19 was confirmed by the real-time polymerase chain reaction (PCR) for SARS-CoV-2.

**Interventions:**

RDV was initiated within 16 hours after the onset of fever (≥ 37.4°C) or positive PCR result in all HD patients, and continued at 100 mg/day intravenously once daily for either consecutive 5 or 10 days.

**Outcomes:**

All patients fully recovered within 2 weeks and did not develop severe COVID-19. Two patients experienced mild liver dysfunction, but it was temporary and remitted spontaneously even continuing RDV treatment. Discontinuation of RDV therapy due to adverse events was not required in any patients.

**Lessons:**

Present cases indicated early intervention with RDV may contribute the favorable outcome and daily administration of RDV for up to 10 days was well tolerated even in HD patients. Literature review showed no previous article reported the efficacy and safety of such earlier and longer administration of remdesivir as in the present cases, therefore this report is informative for clinicians to consider the usage of RDV in HD patients.

## 1. Introduction

As the virulence of severe acute respiratory syndrome coronavirus 2 (SARS-CoV-2) declined and the antiviral strategy such as vaccine and antiviral drugs developed, mortality rate of coronavirus disease 2019 (COVID-19) has decreased recently.^[1,2]^ However, COVID-19 can be still critical for patients with risk factors including hemodialysis (HD).^[3]^ Patients on HD are particularly vulnerable to severe COVID-19 because they are often older age and have many comorbidities such as hypertension and diabetes. According to large studies conducted before the emergence of the Omicron variant, the mortality rate among HD patients with COVID-19 was 20 to 26%.^[4–6]^ In terms of Japanese HD patients, the mortality was reported to be 31%, which was 20 times higher than that of the general population (1.5%).^[7]^ Although the mortality has declined during the Omicron phase, the rate for HD patients was still high compared to general population.^[8,9]^ Beppu, et al^[8]^ reported the mortality rate for HD patients with COVID-19 was changed from 16% to 4% between pre-Omicron and Omicron phase, thus the rate has decreased but remains high. Additionally, patients receiving in-center HD are always exposed to the risk of SARS-CoV-2 infection and involvement of nosocomial cluster. One study showed that patients receiving in-center HD had an approximately 2-fold greater risk of SARS-CoV-2 infection compared to patients receiving home dialysis.^[5]^

For COVID-19 patients with risk factors, antiviral therapy is recommended to avoid disease progression; remdesivir (RDV) is considered as the first line drug for hospitalized COVID-19 patients.^[10–12]^ As for the usage of RDV in HD patients, clinical trials of RDV have excluded HD patients for safety reasons. Therefore, the evidence has not been established and accumulation of knowledge on the regimen, efficacy, and tolerability of RDV in HD patients is important. Here, we report 7 cases of HD patients with COVID-19 expeditiously initiated and successfully treated with daily administration of RDV without any significant adverse event. In addition, previous articles reporting the usage of RDV in HD patients were summarized.

## 2. Case presentation

A nosocomial COVID-19 cluster was occurred at the ward for the Department of Cardiovascular, Nephrology, and Neurology in the University of the Ryukyus Hospital, Okinawa, Japan from August 31 to October 12 in 2021. At that time, the Delta variant was predominant in the region. During the cluster, 11 health-care workers and 20 inpatients including 7 HD patients were infected with SARS-CoV-2. The diagnosis of COVID-19 was confirmed by the real-time polymerase chain reaction (PCR). Table 1 shows the background and clinical course of the 7 HD patients. The median age of these patients was 79 years old (range: 54–92) and 2 patients (28%) were male. Except for end-stage kidney disease, hypertension was the most common comorbidity (71%), followed by diabetes (57%). Two patients had cancer and no patient had an underlying respiratory disease. As for immunosuppressive agent, 2 patients were taking prednisolone (20 mg/day and 15 mg/day, respectively) for the treatment of their primary diseases.

**Table 1:** Patient background and clinical course.

	Patient 1	Patient 2	Patient 3	Patient 4	Patient 5	Patient 6	Patient 7
Patient background							
Age/sex	79/male	92/female	82/male	90/female	67/female	74/female	54/female
BMI	21.5	23	21.2	21.1	17.3	23	19.5
Comorbidities	DM, HTN, renal cancer	DM, HTN, ANCA-AAV	DM, HTN	DM, HTN, PAD, breast cancer	CHF, gastric ulcer	IE, HTN	SLE
Duration of HD	<1 mo	<1 mo	<1 mo	1 yr	11 yr	4 yr	4 mo
Immunosuppressant	None	PSL	None	None	None	None	PSL, HCQ
SARS-CoV-2 vaccination	None	Once	Twice	Twice	Twice	Twice	None
Clinical course of COVID-19							
Symptoms	Fever	Fever	None	Fever, cough	Fever, cough	Fever, cough, sore throat	Fever, cough
SpO_2_ (%) at onset	99	95	99	97	91	98	98
Oxygen administration	None	None	None	None	1 L/min (NC)	None	None
Chest CT finding	Patchy GGO	None	Patchy GGO	None	None	None	None
Treatment for COVID-19	RDV, casirivimab/imdevimab	RDV	RDV	RDV	RDV	RDV	RDV, casirivimab/imdevimab
Initiation of RDV* (h)	10	10	10	16	−2	1	−6
Duration of RDV (d)	10	10	10	5	10	5	10
Liver dysfunction†	None	Yes	Yes	None	None	None	None
Other adverse events	None	None	None	None	None	None	None
Outcome	Discharged	Discharged	Discharged	Discharged	Discharged	Discharged	Discharged

Normal ranges of AST and ALT at the University of the Ryukyus Hospital were 30 and 23, respectively.

ANCA-AAV = anti-neutrophil cytoplasmic antibody–associated vasculitis, ALT = alanine aminotransferase, AST = aspartate aminotransferase, BMI = body mass index, CHF = chronic heart failure, CT = computed tomography, DM = diabetes mellitus, GGO = ground glass opacity, HCQ = hydroxychloroquine, HD = hemodialysis, HTN = hypertension, IE = infectious endocarditis, NC = nasal cannula, PAD = peripheral arterial disease, PCR = polymerase chain reaction, PSL = prednisolone, RDV = remdesivir, SLE = systemic lupus erythematosus.

* Time from onset (or positive PCR result in Patient 3) to initiation of RDV.

† Elevation of AST and/or ALT more than 2-fold increase of upper limit of normal ranges.

In terms of COVID-19 symptoms, fever (≥ 37.4°C) was seen in 6 (86%) patients, followed by cough (57%). One patient (Patient 3) was asymptomatic. At the time of COVID-19 diagnosis, chest computed tomography (CT) was performed in all patients and patchy ground-glass opacity, typically seen in COVID-19, were observed in 2 patients. One patient (Patient 5) with no pneumonia shadow on CT needed oxygen administration temporarily (1 L/minute by nasal cannula for 24 hours) during she had high fever. Based on the World Health Organization severity definition,^[13]^ all patients were classified as non-severe COVID-19 at the time of COVID-19 diagnosis.

As shown in Table 1, all patients received RDV within 16 hours after the onset of fever (≥37.4°C) or positive PCR result in one asymptomatic patient (Patient 3). RDV was administered at 100 mg/day intravenously once daily for either consecutive 5 or 10 days, depending on the vaccination status and clinical condition. On days coinciding with HD, RDV was administered after the HD session. Two patients (Patients 1 and 7) also received casirivimab/imdevimab. As for side effect of RDV, 2 patients (Patients 2 and 3) had elevation of aspartate aminotransferase (AST) and alanine aminotransferase (ALT), defined as more than 2-fold increase of upper limit of normal ranges, during the treatment of RDV. Patient 2 had the elevation of both AST and ALT (85 and 53 U/L at the maximum, respectively) and Patient 3 had the elevation of ALT alone (86 U/L at the maximum). The elevation of AST and ALT in these patients was temporary and remitted spontaneously even continuing RDV treatment. Discontinuation of RDV therapy due to adverse events was not required in any patients. All patients eventually fully recovered within 2 weeks and did not develop severe COVID-19.

## 3. Discussion

We report 7 cases of HD patients with COVID-19 expeditiously initiated (within 16 hours) and successfully treated with daily administration of RDV without any significant adverse event. No patient developed severe condition or died due to COVID-19 during the cluster. Compared with the pre-Delta time frame, there was an increased risk of hospitalization and death with the Delta variant.^[14]^ At the time of the cluster, the Delta variant was predominant in Japan, as well as other countries. Hence, the presented cases had a high risk of developing severe COVID-19 and early intervention with RDV might contribute the favorable outcome.

RDV, a nucleotide analog that inhibits viral RNA-dependent RNA polymerase, has been approved by the US Food and Drug Administration for the treatment of COVID-19 in May 2020.^[15]^ Since the highest viral load and infectivity of SARS-CoV-2 are observed within the first 5 days from symptom onset,^[16]^ early administration of RDV is critically important and guidelines recommend to giving RDV early in the course of COVID-19.^[10–12]^ However, the evidence regarding the usage of RDV in HD patients is lacking because clinical trials have excluded HD patients for safety reasons. Given this background, accumulation of clinical experience is important. A nationwide cohort study targeting Japanese HD patients with COVID-19 reported lower mortality in patients receiving RDV.^[7]^ Although there are some case reports describing the usage of RDV in HD patients, few articles discussed the importance of early administration of RDV. Shah et al^[17]^ compared the length of hospital stay between patients received RDV on day 3 to 6 of illness (N = 23) and patients received RDV on day 7 to 10 of illness (N = 16). As a result, duration of hospitalization was lesser in the former group (8.8 vs 10.7 days) though the difference was not statistically significant. On the other hand, Aiswarya et al^[18]^ reported that administration of RDV within 48 hours after admission in HD patients shortened the mean duration of hospitalization by 5.5 days (*P* = .001) compared with those received RDV after 48 hours. However, it is noteworthy that the mortality rate in the early administration group was still high (21%).^[18]^ Considering the high mortality in HD patients with COVID-19, it is critical to administer antiviral drugs as soon as possible. Our report suggests that extremely early administration (within 16 hours) contributed to inhibit progression of COVID-19, with no severe or dead cases. Although the number of patients was small, our case report reminds us of the importance of early administration of RDV for patients with risk factors.

When this nosocomial cluster occurred, the use of RDV in patients with renal impairment was under debate because RDV can cause hepatic and renal toxicity due to accumulation of sulfobutylether-β-cyclodextrin in these individuals. Therefore, it was recommended not to use RDV in patients with renal failure who have an estimated glomerular filtration rate of <30.^[19]^ On the other hand, several reports showed that RDV was well tolerated in HD patients because sulfobutylether-β-cyclodextrin is dialyzable.^[17,18,20,21]^ Consistent with the results of previous studies, there was no significant adverse event in our cases. Current guidelines recommend that RDV should be administered at a dose of 200 mg on day 1, followed by 100 mg daily from day 2, with the total treatment duration ranging from 3 to 5 days based on the severity of the disease.^[10,11]^ The duration may be extended to a maximum of 10 days if the treatment response is poor. Although the tolerability remains unverified in large-scale clinical trials, RDV can be used in patients with renal impairment including HD patients without dose adjustment.^[11]^ At the time of this nosocomial cluster, an administration method of RDV for HD patients had not been established. Consequently, RDV was administered at 100 mg daily without a loading dose of 200mg, based on the findings reported by Aiswarya et al.^[18]^ The duration of RDV administration was mainly determined by the patients’ vaccination status and clinical condition as well as shared decision making. Additionally, given that the Delta variant was prevalent during this period and all patients had multiple risk factors, 71% (5 out of 7) of the patients received RDV treatment for 10 days. To date, no guidelines have delineated the optimal timing of RDV administration in HD patients. In the previous articles, RDV was administered before or after HD on the day of HD, or once daily regardless of the day of HD, however high tolerability was reported regardless of the timing of RDV administration.^[7,18,22–24]^ Although further accumulation of evidence and clinical experience is needed, the presented cases suggest the administration of RDV once daily for consecutive 10 days at maximum is tolerable even in HD patients. As shown in Table 2, no previous article reported the efficacy and safety of such earlier and longer administration of RDV as in the present cases,^[17,18,21–25]^ thus this report is referable for clinicians to consider the regimen of RDV treatment in HD patients. In conclusion, we showed the importance of early administration of RDV in HD patients with COVID-19 and the daily administration of RDV for consecutive 10 days at maximum can be tolerable even in HD patients.

**Table 2:** Articles reporting the usage of RDV in HD patients.

First author	Number of pts with RDV	Period of pts admission	Age	Duration from onset to admission	RDV treatment						Mortality	Notes
					Initiation	Single dose	Timing	Number of doses	Liver dysfunction	Other adverse event		
Zaki et al^[21]^	112 (incl. PD 23)	May 2020–Jan 2021	65 (mean)	ND	Within 2 d after admission in 89% of pts	ND	ND	4 doses (median)	No difference between RDV vs non-RDV	ND	24% (27/112)	RDV-treated pts showed a trend toward improvement in 30-day mortality.
Shah et al^[17]^	39	Jun–Dec 2020	59 (mean)	4.6 d (median)	From day 3 to 10 of illness	200 mg (Day 1), 100 mg (Day 2–4)	ND	Up to 5 doses	Mild in approx. 10% pts	ND	28% (11/39)	Hospitalization was shorter in early-treated pts (day 3–6 vs 7–10 of illness) but no statistical difference
Aiswarya et al^[18]^	48	Jul–Sep 2020	50.1 (mean)	3 d (median)	2 d (median) after admission	2.5 mg/kg (up to 100 mg)	4 h before HD	Up to 6 doses	None	Behavioral disorder in 1 pt	21% (10/48)	Earlier treatment (within 48 h after admission) with RDV resulted in earlier discharge (−5.5 d)
Ito et al^[22]^	7	Jun–Aug 2021	40s–80s	ND	2 d (mean) after admission	100 mg	ND	Up to 5 doses	Mild in 1 pt	None	14% (1/7)	All pts treated with RDV at the diagnosis of moderate COVID-19 were recovered
Lim et al^[24]^	44	Jan–Mar 2022	70.2 (mean)	1.8 d (mean) [Dx to admission]	Within 7 days of onset of symptoms	100 mg (Day 1), 50 mg (Day 2–4)	After HD on days coinciding with HD	Up to 5 doses	No difference between RDV vs non-RDV	ND	2.3% (1/44)	RDV reduced the risk of composite of mortality, high-flow nasal cannula use, and ICU transfer
Cuadrado-Payán et al^[23]^	21	Dec 2021–Mar 2022	71.4 (median)	ND	Within 48 h after COVID-19 Dx	200 mg (Day 1), 100 mg (Day 2–4)	After HD on days coinciding with HD	Up to 5 doses	None	None	8% (3/36)	RDV was well-tolerated but there was no difference on the efficacy compared to control group
Nakaya et al^[25]^	25	ND	78 (median)	ND	ND	100 mg on HD day	4 h before HD	Up to 6 doses	ND	ND	0%	All pts received neutralizing abtibody (Sotrovimab) 48 h after admission
Oshiro et al (this report)	7	Aug–Oct 2021	79 (median)	0	Within 16 h after onset of fever or Dx	100 mg	After HD on days coinciding with HD	5 or 10 doses	Mild in 2 pts	ND	0%	Early intervention with RDV might lead to no death. Daily treatment for 10 days was well tolerable

Dx = diagnosis, HD = hemodialysis, ICU = intensive care unit, incl = including, ND = not described, PD = peritoneal dialysis, pt(s) = patient(s), RDV = remdesivir.

## Author contributions

**Conceptualization:** Nanako Oshiro, Takeshi Kinjo, Kentaro Kohagura.

**Data curation:** Nanako Oshiro, Daigo Aharen, Yuki Kudo, Eisuke Katsuren, Kumiko Omine, Takuto Nakamura, Ryo Zamami, Akio Ishida, Kazuya Miyagi, Masashi Nakamatsu, Kazuko Yamamoto, Kenya Kusunose, Jiro Fujita, Yusuke Ohya.

**Supervision:** Takeshi Kinjo, Kentaro Kohagura.

**Writing – original draft:** Nanako Oshiro, Takeshi Kinjo.

**Writing – review & editing:** Nanako Oshiro, Takeshi Kinjo, Kentaro Kohagura.
